# Selective Vacuum Evaporation by the Control of the Chemistry of Gas Phase in Vacuum Refining of Si

**DOI:** 10.1021/acs.langmuir.1c00876

**Published:** 2021-06-08

**Authors:** Arman Hoseinpur, Stefan Andersson, Kai Tang, Jafar Safarian

**Affiliations:** † Department of Materials Technology, Norwegian University of Science and Technology (NTNU), Trondheim 7034, Norway; ‡ SINTEF Industry, Trondheim 7465, Norway

## Abstract

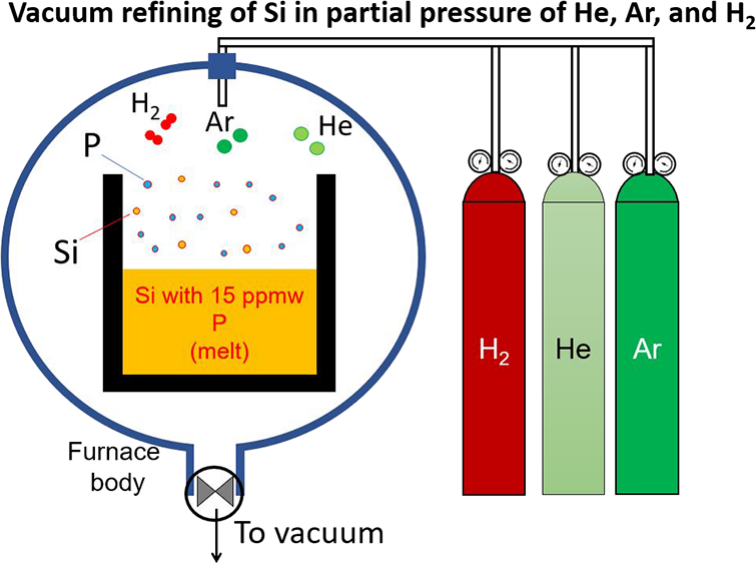

The evaporation of P from liquid Si under vacuum and reduced pressures of H_2_, He, and Ar was studied to evaluate the feasibility of effective P removal with insignificant Si loss. It was found that the introduction of Ar and He inert gases at low pressures reduces the rate of P removal, and their pressure decrease will increase the process rate. Moreover, the kinetics of P removal was higher in He than in Ar, with simultaneous lower Si loss. Under reduced pressures of H_2_ gas, however, the P removal rate was higher than that under vacuum conditions with the lowest Si loss. Quantum chemistry and dynamics simulations were applied, and the results indicated that P can maintain its momentum for longer distances in H_2_ once it is evaporated from the melt surface and then can travel far away from the surface, while Si atoms lose their momentum in closer distances, yielding less net Si flux to the gas phase. Moreover, this distance is significantly increased with decreasing pressure for H_2_, He, and Ar gases; however, it is the largest for H_2_ and the lowest for Ar for a given pressure, while the temperature effect is insignificant. The rate of P evaporation was accelerated by applying an additional vacuum tube close to the melt surface for taking out the hot gas particles before they lose their temperature and velocity. It was shown that this technique contributes to the rate of process by preventing condensing gas stream back to the melt surface.

## Introduction

Vacuum evaporation of metals and alloys has been researched for more than a century and has found a lot of applications in industries such as refining metals to remove volatile species,^1−6^ evaporation of metals from melt pools in physical vapor deposition (PVD) applications,^7^ and so forth. Vacuum evaporation is a strong tool for molten metal purification from impurities that have higher evaporation rates than major elements. The vacuum refining process can be applied to treat steel,^8^ nickel super alloys,^9^ aluminum,^10^ and copper melts.^4^ Solar-grade silicon (SoG-Si) is a special grade of silicon,^11−14^ which is free of impurities (6 N) and has a maximum limit of phosphorus of about 0.1 ppmw. Vacuum refining is recently used for silicon purification to remove phosphorus and is a candidate process in SoG-Si production,^2,3,15,16^ and it is scaled up in pilot production.^17^ Vacuum refining of Si is the most efficient method to remove phosphorus from Si,^3^ and its importance will increase regarding the growth of the photovoltaic (PV) market in the future.

**Figure 1:**
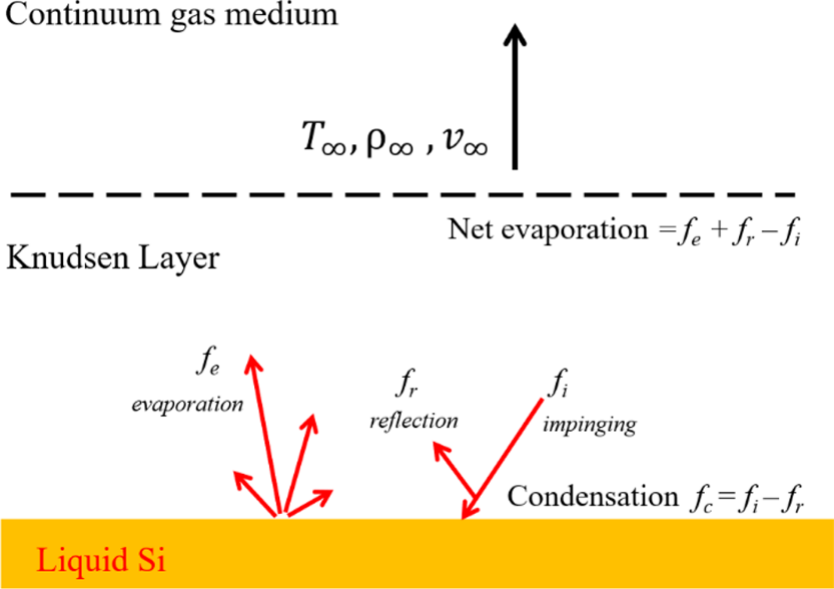
Sketch of evaporation from a liquid surface, including the Knudsen layer and the continuum media.

Vacuum evaporation of liquid metals was first studied and theorized by Hertz in 1882^18^ and Knudsen in 1915,^19^ and they showed that when a matter evaporates into the vacuum, the atoms emitted from the surface travel in cometary trajectories in a thin layer with a thickness of few mean-free paths (λ), without having interactions and moving freely between the collisions. This layer is called the “Knudsen layer,” and the gas particles have a full-range Maxwellian velocity distribution which relaxes to a continuum flow. Figure 1 shows a vacuum evaporation schematic from a melt surface based on the Hertz–Knudsen theory. This figure shows that the evaporation of atoms from the melt surface is a function (*f*_e_) of liquid temperature (*T*_l_), equilibrium vapor pressure (*p_i_^e^*) or molar density (ρ*_i_^e^*) of the evaporating element, and the velocity field (ξ→). In addition to evaporation, Hertz^18^ and Knudsen^19^ reasoned that there is a back flux of atoms to the melt surface, which can be expressed by function (*f_i_*), depending on the gas properties in the continuum region such as temperature (*T*_∞_) and density (*p*_∞_). Only a part of the impinging atoms can condense to the liquid phase and the rest of them will be reflected, which is shown by *f_i_*. Here, we suffice to the introduction of these functions and the phenomena involved in vacuum evaporation, and more details about the mentioned functions can be found in previous studies.^20−22^ Schrage^23^ contributed to this model by taking the continuum medium into account and considered the effect of the mean velocity of outflow gas (*v*_∞_) on the Maxwellian distribution of gas particles in the Knudsen layer, leading to the introduction of parameter Γ. According to Schrage,^23^ when there is no gas velocity Γ = 1 and when *v*_∞_ increases, the value of Γ reduces. As shown in Figure 1, only a part of the molecules impinging the liquid surface absorb and condense, and the rest of the molecules reflex from the surface. Ytrehus and Østmo^20^ investigated the basic fluid flow and added β to eq 1 which represents the nonequilibrium backscattering of gas particles. From the pioneer studies of Hertz^18^ and Knudsen,^19^ many scientists contributed to their work, focusing on modifying the condensation part, and the well-developed version of the Hertz and Knudsen model is presented as follows:
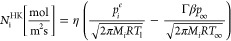
1where *N*_i_^HK^ is the net evaporation molar flux of element *i*, *T*_l_ is the liquid surface temperature, *M_i_* is the molar mass of the element *i*, *R* is the universal gas constant, and *T*_∞_ is the gas temperature in the continuum medium, beyond the Knudsen layer. In eq 1, the first fraction presents the evaporation from the liquid surface and the second fraction presents the condensation from the gas phase to liquid phase. Therefore, both Γ and β deal with the condensation term. Γ and β could be defined in terms of dimensionless velocity called the external speed ratio:^20^
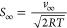
2

The external speed actually represents the ratio of *v*_∞_ (average velocity of the gas) over the thermal velocity () of gas. Safarian^21^ plotted Γ, β, and their product (Γβ) versus *S*_∞_ and showed that by the increase of *S*_∞_, Γβ reduces to a minimum value of 0.53, when *S*_∞_ ≈ 0.5. Schrage^23^ explained that *from the molecules streaming out from the liquid surface, only a fraction* (η) *originates because of spontaneous processes inherent in the surface; for any given state of the surface, there will be a certain number of molecules having the characteristics enabling them to leave the surface and enter the gas phase*. Early experimental measurements of Knudsen^19^ revealed that η has a value close to unity. Knudsen applied effusion out of cells with tiny orifices for developing his theory. However, Langmuir^24^ studied the evaporation from free surfaces^25^ and assumed that at equilibrium and low pressures (<1 mm of mercury), the rates of evaporation and condensation are low, and hence, the evaporation rate can be independent of condensation. Therefore, considering the Langmuir^24^ assumption, under weak evaporation conditions, the condensation term given in eq 1 can be ignored and can be simplified as follows:
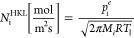
3

This equation is known as the Hertz–Knudsen–Langmuir relation and is mainly applied when dealing with evaporation from free surfaces and liquids with low vapor pressures; it shows the maximum evaporation rate.

The vacuum refining of Si for the removal of phosphorus was studied in the early 1990s,^26,27^ and it was further researched.^8,17,27−35^ Ikeda and Maeda^27^ investigated the phosphorus removal from Si under electron beam melting, Yuge et al.^36^ studied the effect of temperature and Ar partial pressures on the rate of phosphorus removal from Si in an induction furnace, Safarian et al.^2,15^ studied the kinetics of phosphorous removal from Si melt and the mechanism of phosphorus evaporation, including the theoretical modeling of the mass-transfer coefficients for phosphorous diffusion in melt, evaporation from the melt surface, and diffusion through the gas phase. Safarian applied the Hertz and Knudsen evaporation model, presented in eq 1, for calculating the theoretical evaporation rate. The Hertz–Knudsen model for the evaporation of elements from ternary melts whose composition changes intensively during the refining process because of solvent evaporation was recently studied.^16^ Vacuum refining of Si has mainly been carried out in vacuum induction and electron beam furnaces where the melt is stirred vigorously. Then, as already shown,^2^ the overall rate of phosphorus removal from Si will be controlled by the rate of evaporation from the melt surface and diffusion in the gas layer. Therefore, accelerating the process rate should be via faster chemical evaporation at the surface and diffusion in the gas phase. Another issue in the vacuum refining of Si is the undemanding evaporation of Si in this process, which seems to be inevitable. However, the science of evaporation has been under intensive research recently;^11,37−42^ there is no report on a method to make evaporation selective. In this work, we propose a creative method for accelerating the evaporation process, which principally works by reducing the extent of the condensation term introduced in eq 1. In addition, to control Si loss, we have studied the effect of partial pressures of various gases on the selective evaporation of phosphorus from liquid Si.

## Experimental Procedure

Vacuum evaporation of phosphorus from liquid Si was studied in a vacuum induction furnace, and quantum chemistry calculations were applied to interpret the experimental results. The details and methodology of the research are presented in the following sections.

### Materials and Characterization

In most of the experiments carried out in this work, we applied polysilicon (FBR, 8 N purity) as the initial material mixed with 40 wt. % Silgrain (HQ, micron cut; 0.04 wt. % Fe, 0.09 wt. % Al, 0.013 wt. % Ca, 0.001 wt. % Ti, 25 ppmw P, 30 ppmw B) to provide about 10 ppmw phosphorus in the initial Si melt. In two experiments, we mixed a master alloy of Si containing high P content (314.1 ± 7 ppmw) with polysilicon to provide higher concentrations of phosphorus in melt. The details of master alloy production have already been elaborated.^15^ About 400 g of the initial mixture was charged into a high-density graphite crucible (properties are mentioned in a previous study^43^) with an inner diameter of 70 mm and an outer diameter of 85 mm and melted in an induction furnace under vacuum. When performing the vacuum experiments, several samples were taken from the melt at different time intervals to track the phosphorus concentration change during the vacuum induction refining (VIR) process. We elaborated on the sample taking process previously.^3,16^ The samples taken from the melt were digested in a mixture of HF and HNO_3_ acids and then characterized by inductively coupled plasma mass spectroscopy (ICP-MS, Agilent 8800 ICP-MS Triple Quad). In order to study Si solvent evaporation, we attempted to determine the weight change of the crucibles by measuring the weight of the crucible before and after the experiment. However, many crucibles broke during the solidification of Si because of Si expansion upon solidification. However, those crucibles that survived from the solidification were weighed after the experiment, and the silicon evaporation flux was calculated by dividing the silicon mass loss over time.

### Vacuum Refining Setup

The experiments were carried out in a vacuum induction furnace with a chamber volume of 0.5 m^3^ equipped with a mechanical pump that can reach 0.5 Pa without having any sample in the chamber. In this research, various partial pressures under 70 Pa were attempted, and the applied vacuum pump exhibited a pumping rate of 450–400 L/min over the pressure range of 1–70 Pa. Over the period of the experimental work, the furnace chamber was tested for the leak rate before starting the experiments and afterward, obtaining a leak rate of 0.25 Pa/min. The schematic of the furnace is represented in Figure S1 of the Supporting Information. The crucible was wrapped in graphite felt and mica sheet for safety reasons and was then put in the helical coil of the furnace. During the experiments, the temperature of the melt was measured using a thermocouple (type C) submerged in the melt and connected to a data logger. The submerged thermocouple was inserted in an alumina sheath and then in a graphite sheath to be protected from the melt and carbon attack. In addition to the submerged thermocouple, we used an elevating thermocouple (type C) that can move up and down, and it was used for measuring the temperature of the gas phase. This thermocouple was kept at various heights from the melt surface in different experiments to measure the temperature profile in the gas phase from the melt surface to the top of the crucible. The surface area of the melt (*A* = 0.00366957 m^2^) and the surface over volume ratio (*A*/*V* = 22.64 m^–1^) were maintained consistent in all the experiments. Before performing the experiments, the chamber was vacuumed completely to 5 Pa, and then, it was filled with argon (Ar, 6 N), Ar–5% H_2_, or helium (He), depending on the gas to be studied and then vacuumed. This process was repeated three times to make sure there was no air left in the chamber to prevent any melt surface oxidation. Then, it was filled with Ar up to 1 atm, and then, induction heating was switched on. For the experiments investigating the effect of partial pressures on the chamber, we applied the setup configuration presented in Figure S1a of the Supporting Information. In these sets of experiments, the chamber was purged using Ar and He for studying the partial pressure effect of these two gases. While for the experiments studying the H_2_ effect, having the chamber filled with hydrogen was impossible because of safety issues; then, we purged and filled the chamber with the Ar–5% H_2_ gas mixture (6 N) and performed melting in this gas atmosphere. Subsequently, when the Si in the graphite crucible was fully melted, a sample was taken from the melt, and then, the chamber was vacuumed. At this point, the demanding gas in each experiment (H_2_, He, and Ar) was blown into the chamber through mass flow meter (MFC, ALICAT) devices, while the vacuum pump was allowed to work continuously. Once the Si in the crucible was completely melted, a sample was taken to measure the initial concentration of the melt before starting the vacuum refining experiments. Then, the temperature was set to the target temperature of the study, and the chamber was then vacuumed. Then, the partial pressure in the chamber was adjusted by controlling the gas flow rate to the chamber. Depending on the partial pressure required in the chamber, the flow rate was between 0.005 and 0.2 standard liter per minute. In some experiments, the effect of continuous vacuuming conditions with an auxiliary vacuum tube over the melt was studied, and the furnace configuration is shown in the Supporting Information, Figure S1b. This tube was connected to the chamber vacuum pump. A quartz tube with an inner diameter of 10 mm and a molybdenum tube with an inner diameter of 8 mm was applied as the vacuum tubes.

### Quantum Chemistry Calculations

The interactions and dynamics of P and Si atoms with the Ar, He, and H_2_ gases were studied by a combination of quantum chemistry calculations and kinetic theory. The interaction potentials between pairs of species, that is, P–Ar, P–He, P–H_2_, Si–Ar, Si–He, and Si–H_2_, were calculated using CCSD(T) (coupled cluster with single and double excitations and a perturbative treatment of triple excitation), with the *aug-cc-pV(Q + d)Z* basis set.^44^ The CFOUR code was used for all CCSD(T) calculations.^45,46^

## Results and Discussion

The phosphorus concentration changes over the time of refining and under various reduced pressures of Ar, He, and H_2_ and the experiments performed using the auxiliary vacuum tubes are presented in the Supporting Information (Table S1). The overall mass-transfer coefficient for phosphorus evaporation, *k*_P_ (m/s), for each experiment is also calculated and included in Table S1. It was shown in previous studies^2,3,47,48^ that the kinetics of phosphorus evaporation from liquid Si can be explained by the first-order reaction kinetic model as follows:
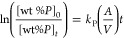
4where [wt % *P*]_0_ and [wt % *P*]*_t_* denote the initial and instant concentrations of phosphorus in the melt, respectively.

### Rate of P Evaporation and Kinetic Parameters

**Figure 2:**
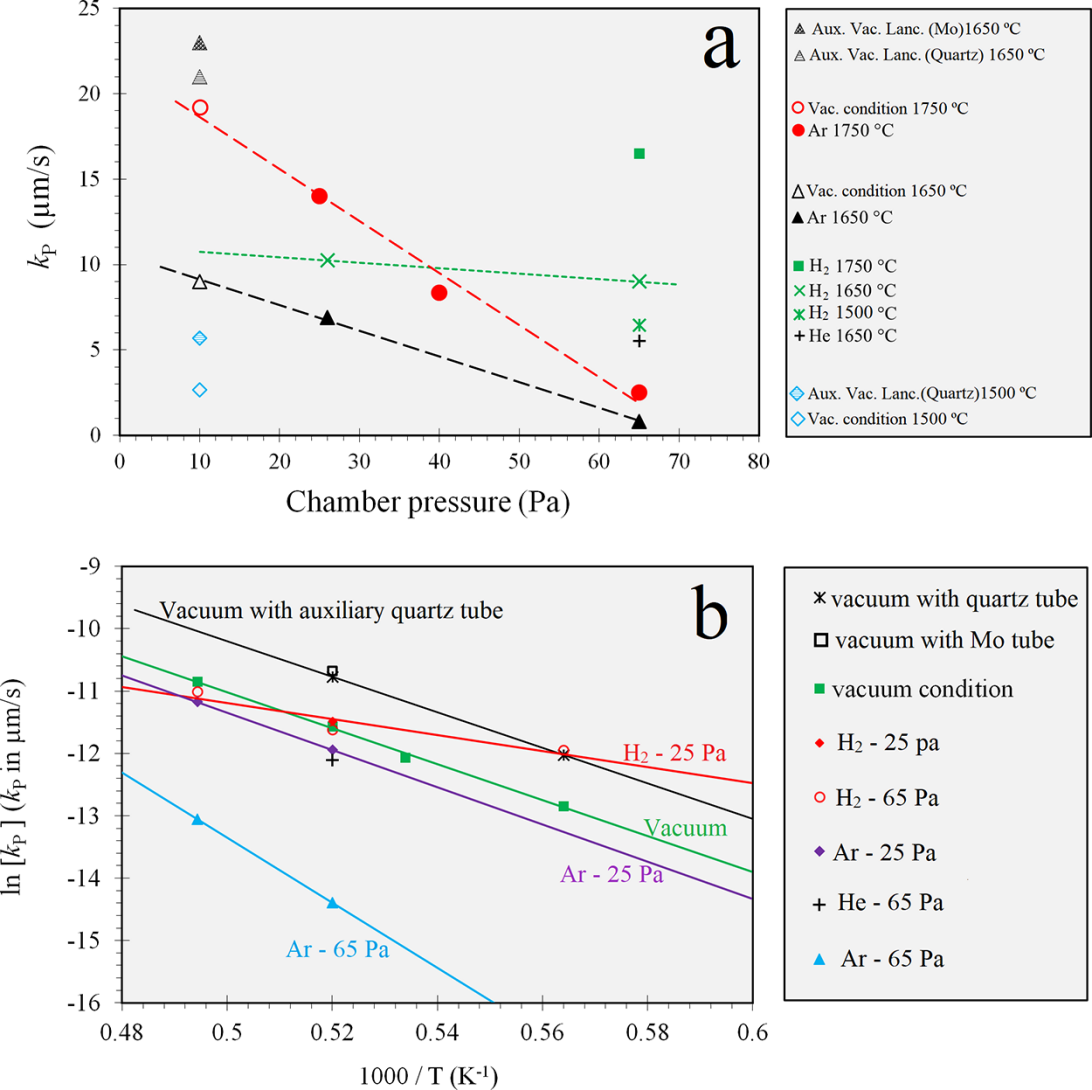
(a) Obtained mass-transfer coefficients as a function of the chamber pressure under reduced pressures of Ar and H_2_. (b) Dependency of *k*_P_ on temperature under various experimental conditions.

Figure 2a represents all the determined *k*_P_ values for various experiments. As seen in Figure 2a, the partial pressure under vacuum conditions (with no gas purging) approaches 10 Pa after 100 min. Therefore, the *k*_P_ values for these experiments were added at 10 Pa. Considering the experiments carried out under reduced Ar pressures, it can be seen that when the pressure of Ar increases in the chamber, *k*_P_ reduces intensively. Considering the data from the vacuum experiments (the data points with no fill), a linear fit line through the *k*_P_ values at each temperature reads the *k*_P_ of the experiment with vacuum conditions. However, Figure 2a shows that the *k*_P_ dependence on pressure for the reduced pressures of H_2_ is less than that for Ar. Considering Figure 2a, it can be seen that *k*_P_ at 65 Pa H_2_ is 11.25 and 7.76 times greater than 65 Pa Ar at 1650 and 1750 °C, respectively. This reveals that H_2_ compared to Ar has a specific effect on the vacuum evaporation rate of P from liquid Si, and H_2_ provides better phosphorus removal conditions. In addition, *k*_P_ for reduced H_2_ pressures changes with a mild slope, and the extrapolated line hits the vacuum condition at the same temperature of 1650 °C, the same as that in the experiments with reduced pressures of Ar. The comparison of experiments 65 Pa H_2_ at 1650 and 1500 °C with 65 Pa Ar at 1750 °C shows that at pressures >40 Pa, the *k*_P_ in H_2_ is greater, even though the temperature is about 100 and 250 °C lower, respectively. In addition, Figure 2a indicates that under the reduced pressure of He, *k*_P_ is higher than that under the same pressure of Ar. However, in comparison with H_2_, He did not show a positive effect as hydrogen showed on the *P* removal rate.

The dependency of *k*_P_ on temperature can be studied by applying the Arrhenius equation as follows:
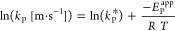
5where *k*_P_^*^ is a constant called the preexponential factor, *E*_P_^app^ is the apparent activation energy for the evaporation of P from Si in J.mol^–1^, and *T* denotes the absolute temperature in Kelvin. By plotting the ln(*k*_P_) values at various temperatures versus the reversed absolute temperature (*T*^–1^) and fitting a line through the data, we can obtain the *E*_P_^app^ and *k*_P_^*^ parameters. Figure 2b shows the Arrhenius curves in case of phosphorus removal in 65 Pa H_2_, 65 Pa Ar, and 25 Pa Ar vacuum condition^3^ and vacuum tube conditions. Obviously, for the higher partial pressure of Ar in the chamber, the Arrhenius lines for phosphorus evaporation are in the lower position. The *k*_P_ for the vacuum condition and 25 Pa Ar show less dependence on temperature than *k*_P_ for 65 Ar Pa. The line of 65 Pa H_2_, however, behaves differently, and it shows the lowest dependence on temperature. It should be mentioned here that the slope of these lines gives the overall activation energy (*E*_P_^app^) for phosphorus evaporation. Figure 2b also reveals that at *T* < 1650 °C and when having 60 Pa H_2_ in the chamber, the *k*_P_ values are 2.4 times those under the vacuum condition. The determined activation energy and the intercept of each curve are presented in Table 1. Considering the data presented in this table, it can be seen that phosphorus removal from the Si melt under vacuum conditions exhibits an activation energy of 239.47 kJ·mol^–1^, and when the experiment is carried out with the vacuum quartz tube, it slightly reduces to 236.70 kJ·mol^–1^. In addition, increasing the partial pressure of Ar in the chamber, the activation energy increases to 248.15 and 433.6 kJ·mol^–1^ for 25 Pa and 65 Pa pressures of Ar, respectively. As mentioned above, hydrogen behaves differently and exhibits a lower *E*_P_^app^ of 107 kJ·mol^–1^, which is 2.25 times smaller than the *E*_P_^app^ under vacuum conditions, and it is attributed to a less *k*_P_ dependency on temperature compared to the Ar presence.

**Table 1:** Determined Apparent Activation Energies and Arrhenius Prefactors for Phosphorus Evaporation from Liquid Si under Different Conditions

research	experimental conditions	*E*_P_^app^ (kJ·mol^–1^)	ln[*k*_P_^*^] (*k*_P_^*^ is in m·s^–1^ )
this work	vacuum with the vacuum quartz tube	236.70	4.036
Hoseinpur & Safarian^3^	vacuum	239.47	3.389
this work	25 Pa Ar	248.15	3.57
this work	65 Pa Ar	433.6	12.73
this work	65 Pa H_2_	107.13	–4.74

In principle, the lower activation energy indicates a smaller energy barrier for the process to proceed, and obviously, the application of the reduced pressure of H_2_ provides better conditions than Ar. Although H_2_ gas is a reactive gas at applied high temperatures, the formation of significant P-containing gaseous compounds is not possible from a thermodynamics point of view under the process conditions. Hence, the lower activation energy for P evaporation under H_2_ (compared to Ar and high vacuum conditions) may not be due to the change in the reaction mechanism via different gaseous species formation. However, hydrogen may affect the melt surface properties, such as surface tension, if it is highly adsorbed at the surface, with an unknown mechanism that accelerates the chemical evaporation mechanism. No literature was found about this case, and the authors could not find supportive theories. Hence, it was assumed that the change in the type of gas in the vacuum chamber does not affect the kinetics of the chemical evaporation of P at the melt surface; therefore, further focus was on the mass-transport phenomena in the gas phase.

### Si Evaporation

**Figure 3:**
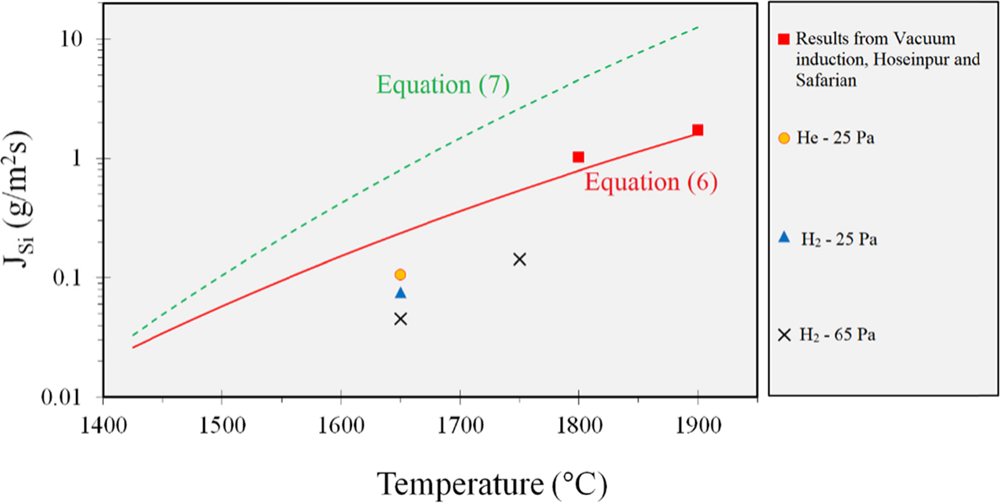
Flux of Si evaporation under vacuum conditions and various experimental pressures, and the maximum theoretical evaporation flux calculated using eqs 6 and 7.

As mentioned in the Introduction section, Si evaporation is undemanding in the vacuum refining process, and it is of interest to find a way to control it. The Si evaporation flux in graphite crucibles and in vacuum induction furnaces was measured by Yuge et al.:^26^
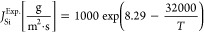
6where *J*_Si_^Exp.^ and *T* denote the experimental flux of Si evaporation and absolute temperature (K), respectively. The theoretical evaporation of Si under a perfect vacuum condition can be calculated using the HKL model already presented in eq 3, and it is presented here again in the form of mass flux, written for Si as follows:
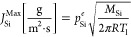
7where *M*_Si_ is the molar mass of Si, and *p*_Si_^*e*^ denotes the Si saturated vapor pressure. Here, we apply both eqs 6 and 7 to calculate the flux of silicon evaporation at various temperatures, as shown in Figure 3. Obviously, the experimental results in our previous experiments^3^ are in good agreement with those reported by Yuge et al.^26^Figure 3 shows that the application of H_2_ and He at reduced pressures leads to a decrease in Si loss. Among the experimental data presented in Figure 3, the case of 65 Pa H_2_ at 1650 °C shows the minimum Si evaporation flux (and hence Si loss) compared to the other experiments, and 3 times lower rate than that shown in eq 6 is observed. In addition, the experimentally measured Si evaporation flux is always smaller than that eq 6 predicts. This was expected because the perfect vacuum condition was not provided in the experimental condition, and hence, the condensation term in eq 1 should not be ignored. However, here, we will introduce another error affecting the Si loss to be calculated from measuring the weight loss of the graphite crucibles in vacuum experiments. The photographs of the crucibles cross sections are also available in the Supporting Information, providing proofs for the above discussion.

### Effect of Vacuum Tubes

**Figure 4:**
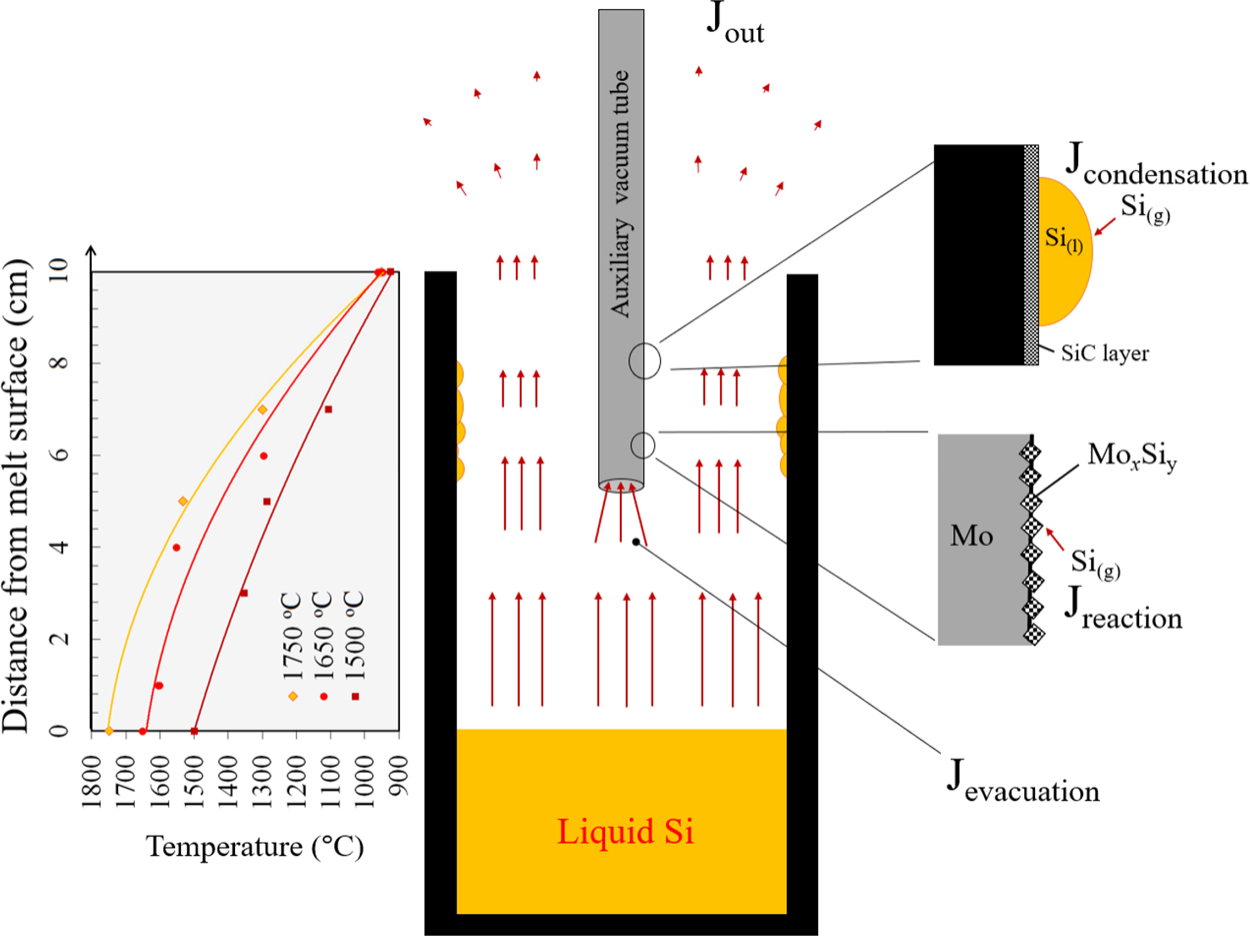
Sketch of the evaporation and condensation phenomena in the crucible with a vacuum tube. The measured gas temperature profile from the melt surface to the crucible edge in different experiments is also shown.

As reported in Table S1 in the Supporting Information, the *k*_P_ using the quartz and Mo vacuum tubes was almost 2 and 2.4 times of the *k*_P_ under the vacuum condition at 1650 °C using the quartz and Mo tubes, respectively. The photograph of the part of the tubes located in the crucible is shown in the Supporting Information, indicating the interaction of the Mo tube with Si vapors evaporating from the liquid Si. The Mo tube tip can interact with the Si vapors to form molybdenum silicides, such as Mo_5_Si_3_, MoSi_2_, and Mo_3_Si. The formation of these silicides from the interaction of Mo with Si_(g)_ is thermodynamically favorable because of their negative Gibbs energy value, as calculated using the HSC software and presented in the Supporting Information. The SEM images of the Mo tube tip are shown in the Supporting Information, and the energy-dispersive X-ray spectroscopy (EDS) characterization indicates the formation of the molybdenum silicide phases on the wall of the Mo tube. Figure 4 illustrates the phenomena taking place during the vacuum evaporation in the crucible, introducing four mechanisms for the removal of the evaporated gas phase as follows:

i. Outflow of the gas phase from above the crucible (*J*_out_).
ii. Condensation of Si on higher parts of the crucible (*J*_condensation_).
iii. Evacuation to the vacuum tube (*J*_evacuation_).
iv. The reaction on the surface of the Mo tube (*J*_reaction_).

The condensation of Si droplets on graphite is well discussed in our previous study.^43^ When the vacuum tube is not applied, the only mechanisms for removing the gas phase (which is mainly Si vapor) from the system are mechanisms *i* and *ii*, and by applying the quartz tube, the vacuum mechanism (*iii*) will be added, and when molybdenum tube is applied all the mechanisms *i* to *iv* come to account. Considering Figure 1, The important role of the above mechanisms is to prevent the condensation of the evaporated gases back onto the melt surface and hence acceleration the evaporation process. In other words, when we have the tubes, the number of atoms impinging the melt surface decreases, and it increases the net evaporation, as shown in Figure 1. The temperature of the gas phase inside the crucible measured using the top thermocouple at different positions is also shown in Figure 4. As shown in this figure, the measurement is carried out for three different liquid Si temperatures, and it is obvious that the temperature of the gas phase decreases, moving away from the melt surface. Figure 4 also indicates that there is a steeper temperature gradient when the melt temperature is higher. In a typical vacuum evaporation experiment, without the vacuum tube, the gas phase in the crucible needs to flow upward and exit the crucible. The higher temperature of the gas phase causes higher gas velocity. Therefore, moving toward the top of the crucible, the gas molecules lose their velocity, and as they exit the crucible, the temperature of the gas decreases immediately. Hence, when the gas is out of the crucible and in the chamber space, it loses its kinetic energy, leading to the reduction of the average velocity of the bulk gas, and it takes a longer time to be evacuated through the main vacuum gate of the furnace chamber (shown in Figure S1 of Supporting Information).

The chamber pressure recorded in the vacuum experiments with and without the quartz tube can also be found in the Supporting Information. The chamber pressure was almost the same and independent of applying the quartz tube. However, as measured, the pressure in the vacuum tube was always lower (about half) than the chamber pressure. Therefore, the gas could be evacuated directly to the auxiliary vacuum tube, before losing its velocity. As explained in the Introduction section, the evaporation flux from the liquid surface is a function of liquid properties, and the vacuum tube cannot affect the evaporating stream of the molecules. However, the evacuation of a part of the gas in the crucible using the vacuum tube prevents the gas from moving back to the liquid surface. In other words, in the presence of the vacuum tube, there is a lower flux of the impinging molecules (*f_i_*) toward the liquid surface, leading to a reduction of the condensation term in eq 1, which subsequently increases the net evaporation from the liquid surface.

**Figure 5:**
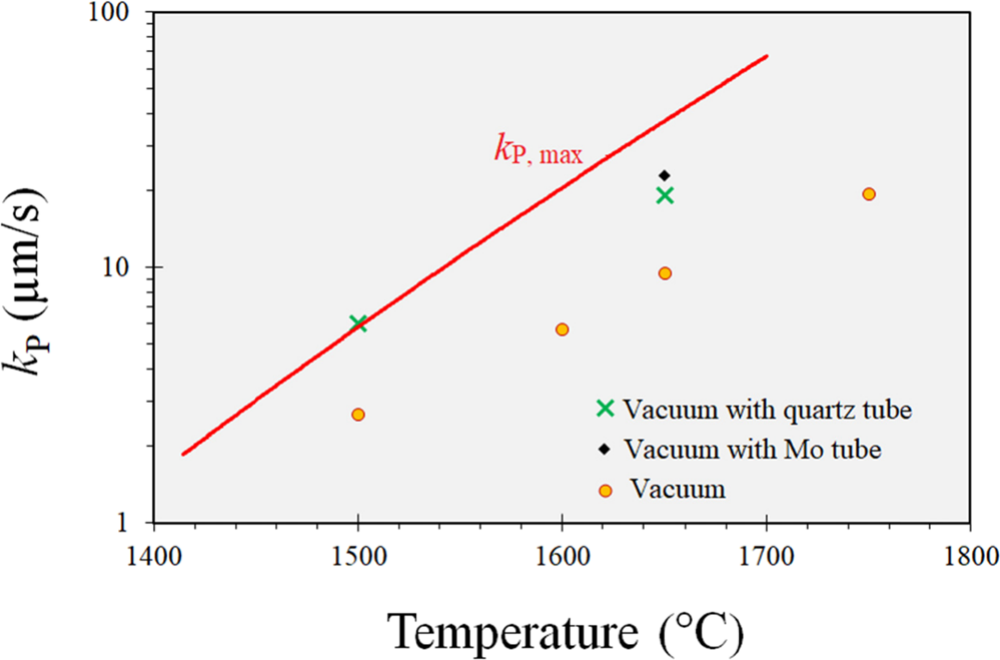
Overall mass-transfer coefficients for phosphorus removal (*k*_P_) under various experimental conditions and the maximum mass-transfer coefficient for evaporation from the melt surface (*k*_P,max_), calculated using eq 9.

In principle, three main steps for phosphorus evaporation from liquid Si are usually considered. (*i*) P mass transport in the bulk melt to the melt surface; (*ii*) chemical evaporation at the melt surface; and (*iii*) mass transport in the gas phase. Hence, the total mass-transfer coefficient (*k*_P_) is as follows:
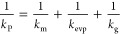
8where *k*_m_, *k*_evp_, and *k*_g_ are the mass-transfer coefficients for steps (*i*) to (*iii*), respectively. In an inductively stirred melt, *k*_m_ will have an extremely high value, and then, *k*_P_ will be mainly determined by *k*_evp_ and *k*_g_, as indicated previously by Safarian and Tangstad.^2^ The maximum mass-transfer coefficient for the vacuum evaporation of element *i* can be written as follows:^2^
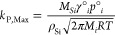
9The calculated *k*_evp_^Max^ over various temperatures is calculated and shown in Figure 5. The results from phosphorus evaporation with and without the vacuum tubes are also presented in this figure. Figure 5 shows that when the vacuum tubes are applied, the overall mass-transfer coefficient reaches *k*_P, Max_ at 1500 °C via removing the resistance for the mass transfer in the gas phase. However, the overall mass-transfer coefficient of 1650 °C is increased using the vacuum tube, however, less than that at 1500 °C. This could be due to more intensive evaporation at the higher temperature and, therefore, slower mass transport through the vacuum tubes under the same pumping rate. This may be supported considering the change of the tube type from quartz to Mo, which shows an even better situation for Mo that has a smaller inner cross-section area (50.2 mm^2^) than the quartz (78.5 mm^2^). For Mo, the significant condensation of Si vapor (and reaction with Mo) can act as a sink for a larger pressure gradient of the Si vapor, yielding a higher mass-transfer coefficient in the gas phase.

### Interactions of Ar, He, and H_2_ Gases with Evaporated P and Si

#### Theory and Computational Details

To investigate the differences in the gas-phase dynamics of evaporated P and Si atoms, formulae derived through the kinetic theory of gases^49,50^ were used. In a dilute gas, an atom with momentum in a given direction will retain a memory of its initial momentum for a given number of collisions with atoms or molecules in the gas, which means that the atom will travel some distance until its motion becomes truly random and independent of its initial momentum. If atoms evaporate in a given direction away from a liquid surface, there will be an average distance traveled along that initial direction until the atomic motion becomes random and normal diffusion behavior determines their mobility. This distance depends on (a) the gas pressure, (b) temperature, (c) the relative masses of the atom and the background gas, (d) the interactions between the colliding species, and (e) the types of collisions in the gas, that is, elastic or inelastic. The mean-free path of a given atom A moving in gas B is given as follows:
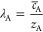
10

where
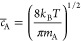
11is the average speed of A with *m*_A_ being the mass of A, and *k*_B_ is the Boltzmann constant.
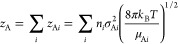
12

is the total number of collisions between atoms A with all other species in the gas. If B is the only other species present, and the concentration of A is much smaller than the concentration of B, this reduces to
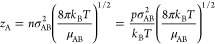
13Here, *n* is the gas number density, *p* is the pressure, and μ_AB_ is the reduced mass of species A and B:
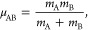
14and σ_AB_ is the distance of the closest approach of two hard spheres with diameters σ_A_ and σ_B_,
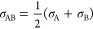
15

After the collision of an atom A with a collision partner B, the component of the velocity of the atom after the collision, ***c****^’^*_A_, parallel to the initial velocity, ***c***_A_, can be written as follows:
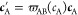
16where ϖ_AB_(*c*_A_) is the persistence ratio for atoms of speed *c*_A._(49) Integrating this quantity over all speeds gives the mean persistence ratio
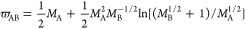
17where *M*_A_ = *m*_A_/(*m*_A_ + *m*_B_) and *M*_B_ = *m*_B_/(*m*_A_ + *m*_B_). For cases where *m*_A_> > *m*_B_, ϖ_AB_ is close to 1, and for *m*_A_ < < *m*_B_, ϖ_AB_ is close to 0. This means that the atoms much heavier than the gas species they encounter will move largely in the same direction through a large number of collisions, whereas relatively light atoms will mostly have changed directions already after a few collisions.

We have combined λ_A_ and ϖ_AB_ to estimate the initial distance traveled by the evaporated atoms, as discussed above. The first collision of an evaporated atom A with a gas species should occur on average after a distance *l*_A_ = λ_A_. By considering a series of collisions of atom A with gas species B, with λ_A_ as the mean distance between each collision and ϖ_AB_ as a measure of the component of the velocity of the atom after collision parallel to the initial velocity in any direction *z* away from the surface, the average distance traveled along *z* can be estimated after two collisions as follows:
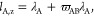
18given that the distance traveled along z is proportional to the velocity component along z. An additional collision adds an average distance z component of ϖ_AB_(ϖ_AB_λ_A_) and so on. Summing up the distances will lead to convergence after a finite number of collisions because 0 < ϖ_AB_ < 1. This sum of distances could be defined as the average distance the atoms move before having lost all memory of the initial momentum and normal diffusion takes over:
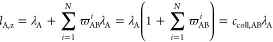
19

The longer the average distance traveled through this mechanism, the higher the probability of the atom not diffusing back to its origin, thereby contributing to the overall effective evaporation of species A.

Distance *l*_A,z_ is related to the Knudsen layer because the derived expression has been arrived at from similar arguments to more advanced theoretical treatments. In the present case, the focus is on the direction of momentum only, whereas in more elaborate studies of the Knudsen layer, the focus is on the relaxation of the initially nonthermal velocity distribution of the evaporating species. Because the focus in our study has not been the nature of the Knudsen layer, *l*_A,z_ should not be taken as the thickness of the Knudsen layer, but it is numerically similar to the theoretical and calculated values of typical Knudsen layer thicknesses (see discussion below). Typical values of the Knudsen layers are 10–20 mean-free paths^20,22,51−53^ or 2–4 *l*_a_,^52^ where *l*_a_ is a typical length scale of Knudsen layers. The relations between *l*_A,z_, *l*_a_, and the mean-free path (λ_A_) are as follows:
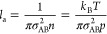
20
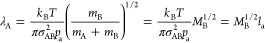
21
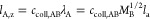
22As can be seen, *l*_A,z_ is inversely proportional to pressure but has a nontrivial dependence on mass through the mean persistence ratio. Numerical values of ϖ_AB_, *c*_coll_, and the ratio of *l*_A,z_ and *l*_a_ are given in Table 2. In addition, it also gives a measure of the average number of collisions needed to randomize the momentum of the evaporating species (*N*_coll_). This is calculated as the number of collisions needed before less than 1% of the initial momentum is likely to remain. For P moving through H_2_ gas, *N*_coll_ = 55, whereas for P in Ar gas, *N*_coll_ = 5.

**Table 2:** Parameters for the Collisions of Evaporating Species A and Gas Species (See Text for Details)^a^

species (A)	gas	σ_high *T*_ /Å	σ_hard sphere_/Å	σ_gas_ /Å	ϖ_AB_	*c*_coll_ = *l*_A, z_/λ_A_	*N*_coll_	*l*_A, z_/*l*_a_
P	H_2_	2.4	3.5	2.915	0.920	12.4	55	3.07
P	He	2.3	3.7	2.576	0.851	6.71	29	2.27
P	Ar	2.8	3.6	3.432	0.342	1.53	5	1.14
Si	H_2_	2.1	N/A	2.915	0.912 / *0*	11.3 / *1.00*	50 / *1*	2.66 / *0.235*
Si	He	2.0	2.9	2.576	0.837	6.15	26	2.40
Si	Ar	2.4	2.9	3.432	0.318	1.47	5	1.12

a For Si–H_2_ collisions, the values in italics refer to the case where collisions are assumed to be completely inelastic.

#### Interaction Potentials

**Figure 6:**
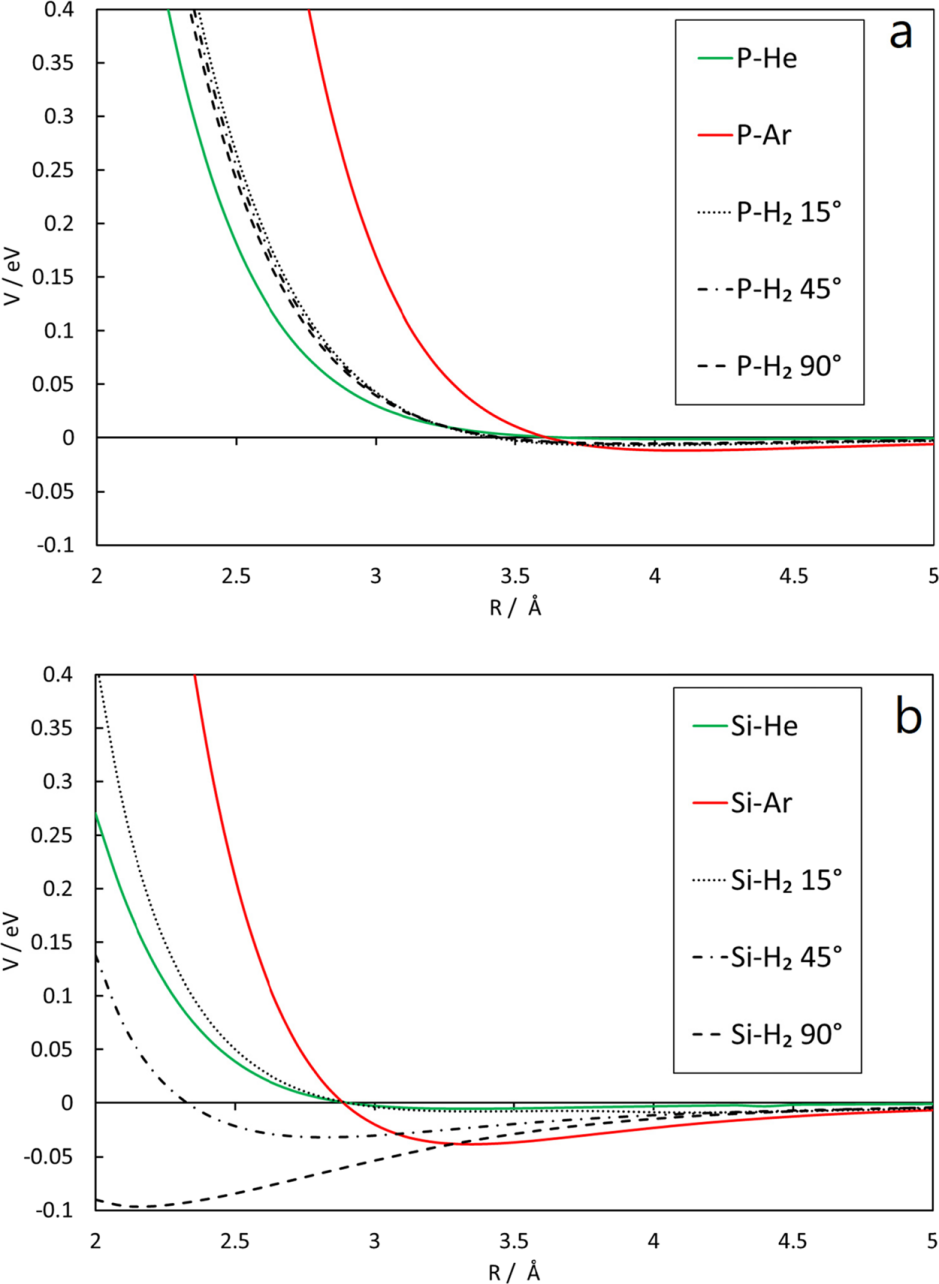
(a) Potential curves calculated using CCSD(T)/aug-cc-pV(Q + d)Z for P–He (green), P–Ar (red) and three angles of approach for P–H_2_ (black): 15° (dotted), 45° (dot-dashed), and 90° (dashed). (b) Potential curves calculated using CCSD(T)/aug-cc-pV(Q + d)Z for Si–He (green), Si–Ar (red) and three angles of approach for Si–H_2_ (black): 15° (dotted), 45° (dot – dashed), and 90° (dashed).

In Figure 6a, the potential curves calculated by CCSD(T) are plotted for the interactions of a ground-state P atom (in the ^4^S electronic state) with Ar, He, and H_2_ gases, respectively. The potential is evaluated at center-of-mass distances between 2 and 5 Å. For the interactions with H_2_, three different angles of approach are considered, that is, 15, 45, and 90°, that are defined as the angle between the H–H bond vector and the vector between P and the center of mass of H_2_. The H_2_ distance is maintained constant, corresponding to the equilibrium distance of 0.742 Å. As can be seen, the potential curves are all repulsive at distances shorter than about 3.5 Å and only weakly attractive at a long range. The three P–H_2_ curves are all very similar, indicating that H_2_ can be reasonably approximated as being spherical, that is, the P–H_2_ potential is near-isotropic. This, together with the repulsive potential, suggests that collisions are likely not strongly inelastic, but to a large extent, elastic. For estimating σ_AB_ to be used in eq 13, the distance where the potential is zero at close encounters could be used. However, this is only valid for low collision energies and would therefore work well around room temperature. To take higher temperatures into account, we have used the classical turning point for a collision energy around 0.3 eV. For P–He, this is approximately 2.3 Å, for P–H_2_, it is 2.4 Å, and for P–Ar, it is 2.8 Å.

The corresponding potential curves for a ground-state Si atom (in the ^3^P electronic state) interacting with Ar, He, and H_2_ are clearly different, Figure 6b. The Si–He and Si–Ar interactions are clearly more attractive at a long range and less repulsive at a short range than the P–He and P–Ar curves, and the Si–H_2_ interaction is clearly quite attractive and strongly anisotropic, being most attractive for a 90° angle of approach. This latter behavior can be understood by realizing that the Si–H_2_ interaction is reactive.^46,54,55^ There is a fairly high calculated energy barrier of about 0.94 eV for forming an excited triplet SiH_2_ molecule.^55^ This triplet SiH_2_ can make spin-forbidden transition to the singlet ground-state SiH_2_ through a crossing point only 0.10 eV above the triplet SiH_2_ minimum.^54^ There is also a predicted crossing between the triplet and singlet potential energy surface upon the approach of Si toward H_2_ at quite a low energy (0.10 eV above Si + H_2_),^55^ which is also a nonadiabatic pathway to form singlet SiH_2_. The spin-forbidden transitions have a probability of the order of 0.1–1% but could nevertheless lead to a substantial reaction. Even in case the Si–H_2_ collision does not lead to the reaction, the attractive and anisotropic interaction should lead to strongly inelastic collisions. The estimated σ_AB_ are 2.0 and 2.4 Å for Si–He and Si–Ar, respectively, reflecting the less repulsive interaction than the case of the P atom. Si–H_2_ collisions will be treated as entirely inelastic, the consequence of which will be discussed below. Detailed dynamics simulations would be needed to accurately treat the collisional behavior of this system, but for the sake of the present study, such simplification should be sufficient for a qualitative discussion.

High-temperature σ_AB_ values estimated in the present study are shown in Table 2 alongside the low-energy hard-sphere σ_AB_ as well as the literature value for σ for the gases under study (H_2_, He, and Ar).^56^

#### Molecular Collision Dynamics

**Figure 7:**
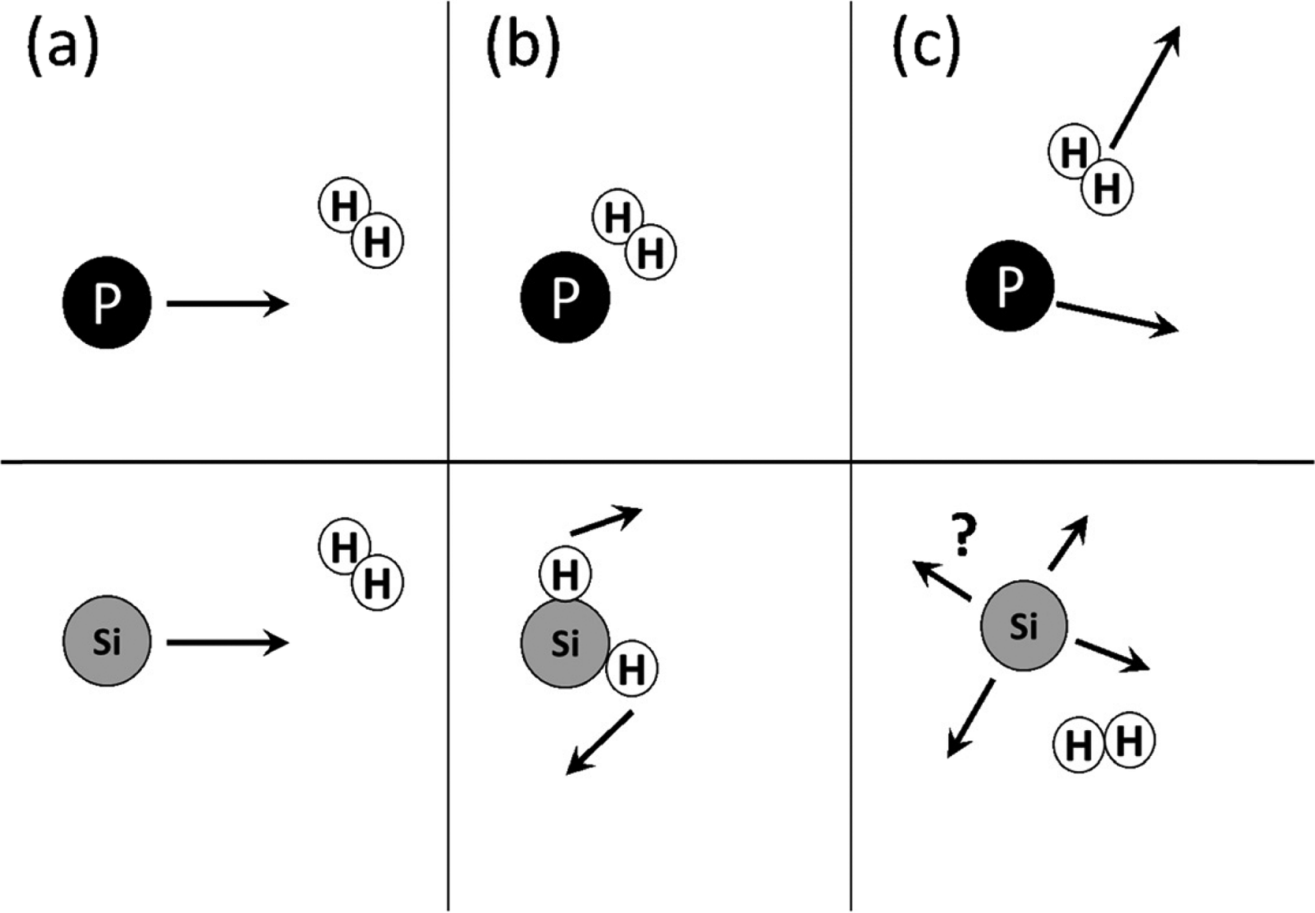
Typical collisions of P and Si with H_2_: (a) initial state, (b) closest encounter, and (c) final state.

As discussed above, the P–H_2_ and Si–H_2_ interactions are qualitatively different, which can be illustrated as shown in Figure 7. The P–H_2_ collisions are likely to be mostly elastic, whereas the attractive interaction of Si and H_2_ has a much higher probability of being inelastic. Equations 17–19 only accurately describe elastic collisions, so a different treatment is needed for Si–H_2_. Recognizing that a collision complex of a given lifetime could be formed in this case because of the attractive interaction, it is reasonable to assume that momenta after the collision are completely random (as illustrated in Figure 7c) and that an effective mean persistence ratio would be close to zero. In the case of Si–H_2_, eq 18 is therefore simplified as follows:
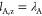
23

#### Nondiffusional Mass Transport

**Figure 8:**
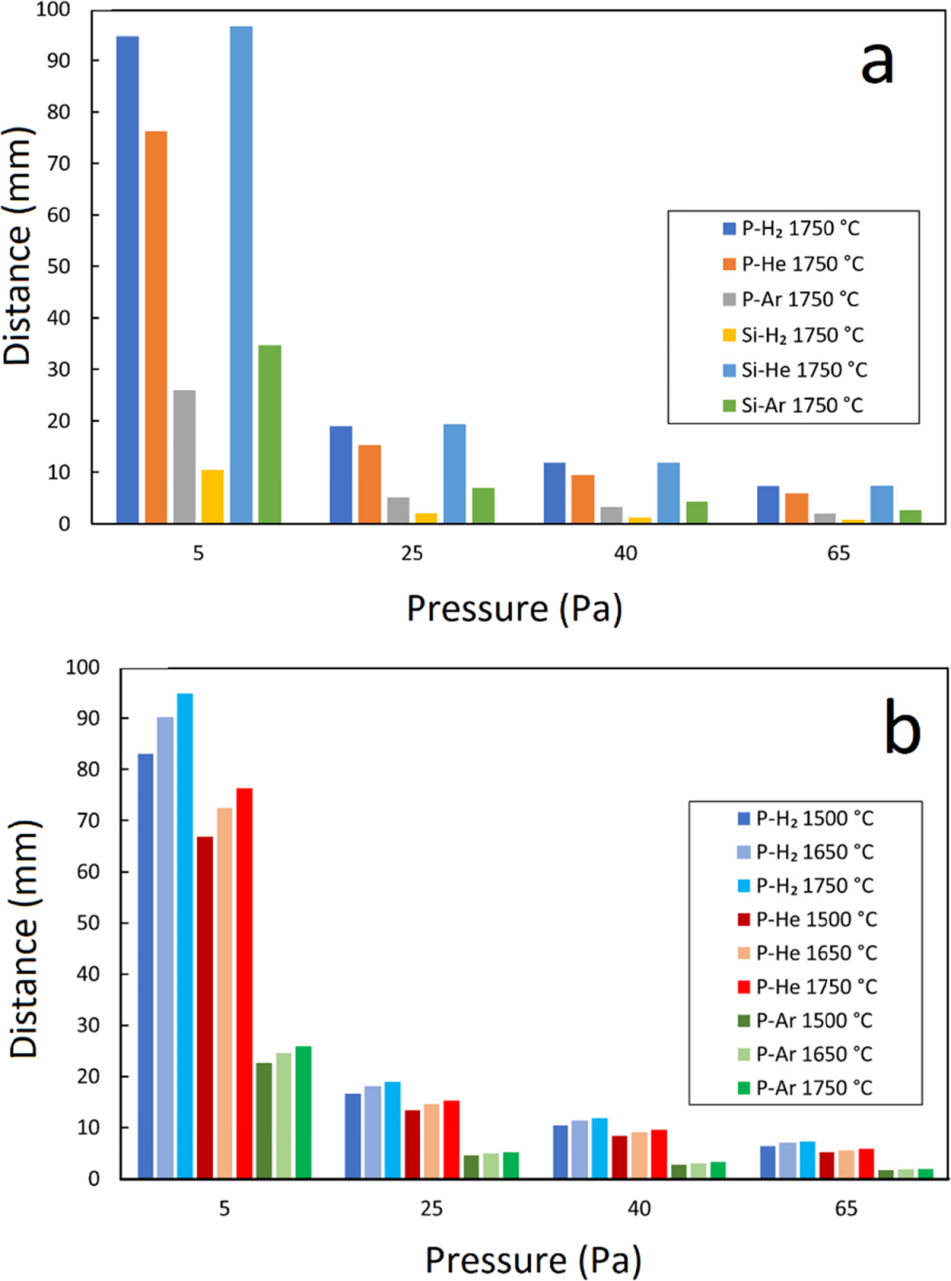
(a) Distances traveled as defined by eqs 18 and 19 by P and Si atoms through various gases at pressures of 5–65 Pa and *T* = 1750 °C and (b) traveled distances for P atoms at *T* = 1500–1750 °C.

**Figure 9:**
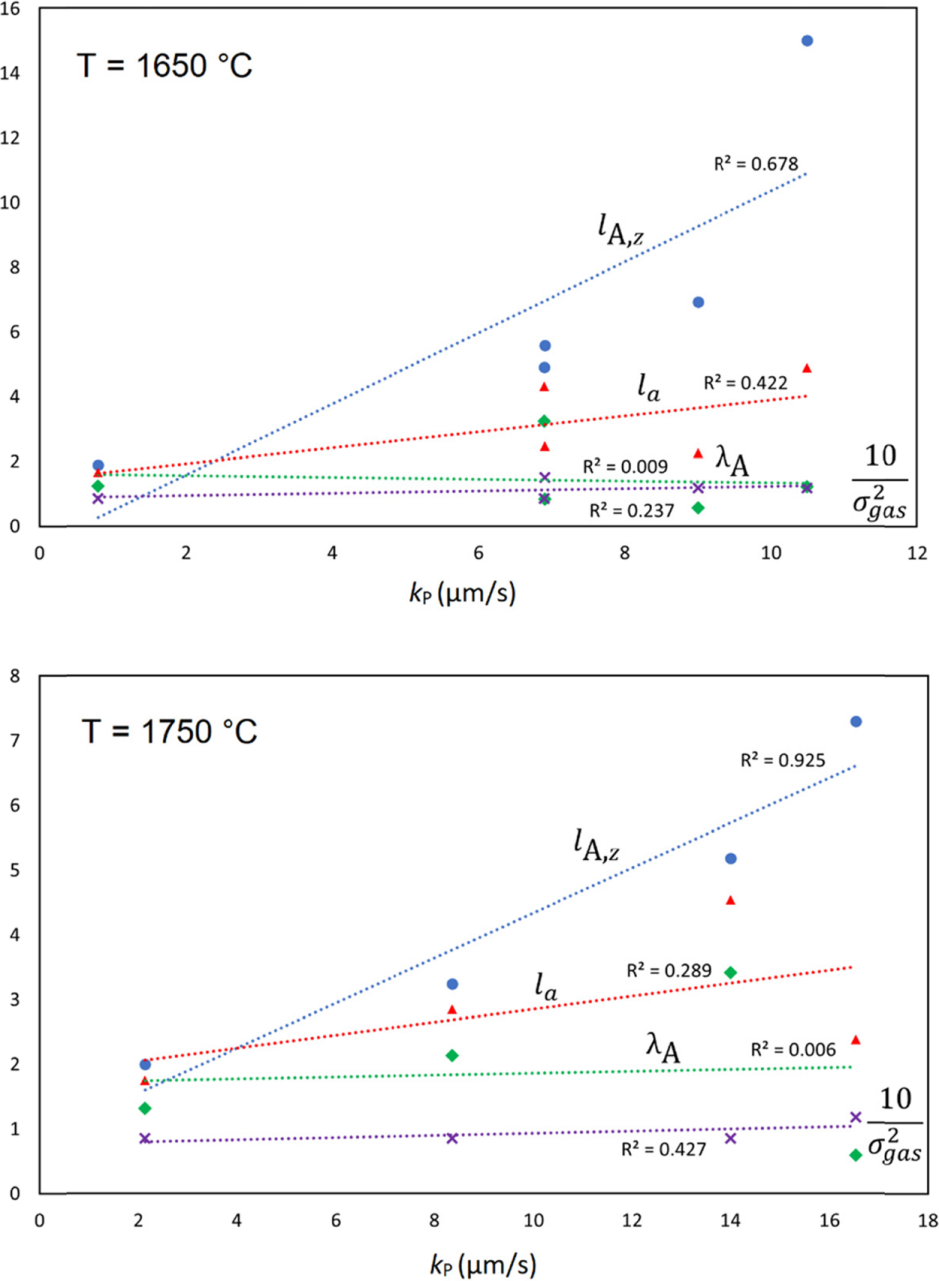
Correlations between measured *k*_P_ and calculated parameters *l*_A,z_, *l*_a_, and λ_A_ as well as literature σ_gas_ for P evaporation at 1650 °C (top) and 1750 °C (bottom).

Figure 8a shows the average initial distances traveled upon evaporation, *l*_A,z_, calculated using eqs 18 and 19 at varying pressures and a temperature of 1750 °C. This indicates that the most efficient evaporation of P should take place through H_2_ gas, and Si most efficiently travels through He. In addition, *selectivity* for P over Si atom transport is only seen in the case of H_2_ because the inelastic collisions of Si in H_2_ efficiently hinder this initial transport away from the surface. In the case of He and Ar, transport of Si seems to be more efficient because of the less repulsive interactions, as discussed above. The temperature dependence of the *l*_A,z_ of P atoms in various gases at a few different pressures is shown in Figure 8b. There is no strong effect of temperature on this transport mechanism itself, see eqs 20 and 22. The values of *l*_A,z_, *l*_a_, and λ_A_ are given in Table 3. Potential correlations between these quantities, as well as the literature value of σ_gas_ given in Table 2, with measured mass-transfer coefficients (Table S1), have been evaluated for P evaporation at 1650 and 1750 °C and are shown in Figure 9. The correlation between *k*_P_ and *l*_A,z_ is good to excellent, whereas the other parameters do not show the same clear correlation with *k*_P_. There is actually zero correlation between the mass-transfer coefficient and the mean-free path, indicating that it is necessary to consider the gas-phase dynamics of vacuum evaporation in order to understand the process. This seems to indicate that *l*_A,z_ could be a good descriptor of the efficiency of vacuum refining under different conditions.

**Table 3:** Parameters for Species Transport in Gases at Different Temperatures and Pressures^a^

evaporating species (A)	gas	*T/ °*C	*p* / Pa	λ_A_ / mm	*l*_A, z_ / mm	*l*_a_ / mm
P	H_2_	1500	25	1.34	16.6	5.41
P	H_2_	1500	65	0.514	6.39	2.08
P	H_2_	1650	5	7.25	90.1	29.3
P	H_2_	1650	25	1.45	18.0	5.87
P	H_2_	1650	65	0.558	6.93	2.26
P	H_2_	1750	25	1.53	19.0	6.17
P	H_2_	1750	65	0.587	7.29	2.37
P	He	1650	5	10.8	72.5	32.0
P	He	1650	25	2.16	14.5	6.39
P	He	1650	65	0.831	5.58	2.46
P	Ar	1650	5	16.2	24.6	21.6
P	Ar	1650	25	3.24	4.92	4.31
P	Ar	1650	65	1.24	1.89	1.66
Si	H_2_	1500	25	2.09	20.7 / *1.83*	7.79
Si	H_2_	1500	65	0.705	7.97 / *0.705*	3.00
Si	H_2_	1650	5	9.94	112.4 / *9.94*	42.3
Si	H_2_	1650	25	1.99	22.5 / *1.99*	8.45
Si	H_2_	1650	65	0.764	8.65 / *0.764*	3.25
Si	H_2_	1750	25	2.09	23.6 / *2.09*	8.89
Si	H_2_	1750	65	0.804	10.1 / *0.804*	3.42
Si	He	1650	5	14.9	91.9	38.3
Si	He	1650	25	2.99	18.4	7.67
Si	He	1650	65	1.15	7.07	2.65
Si	Ar	1650	5	22.5	33.0	29.3
Si	Ar	1650	25	4.50	6.60	5.87
Si	Ar	1650	65	1.73	2.54	2.26

a See the Text for Details.

**Figure 10:**
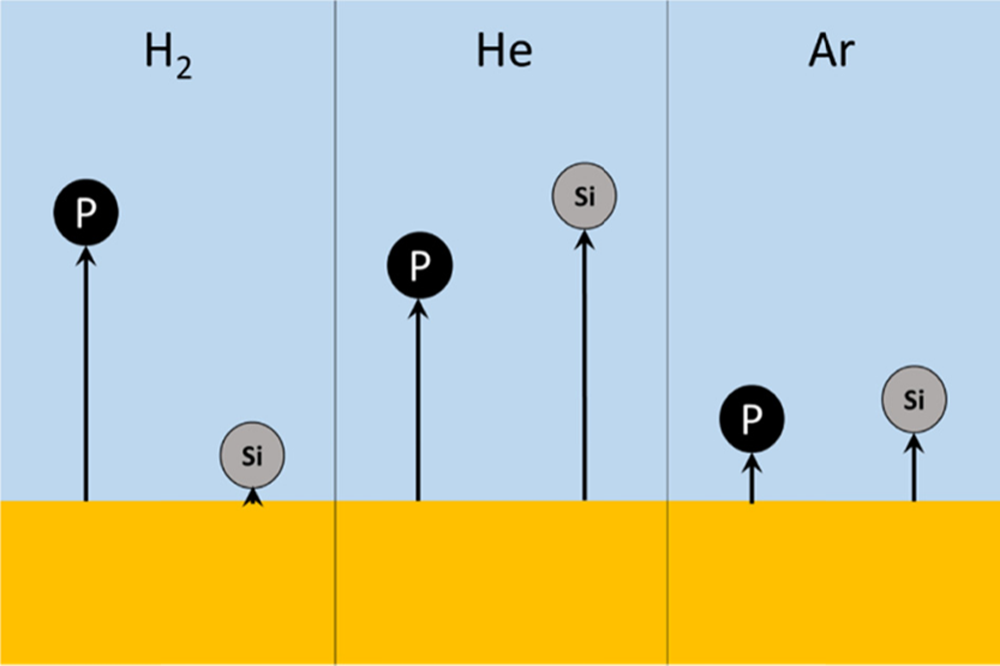
Illustration of initial nondiffusional transport of P and Si atoms from a melt surface in low pressures of H_2_, He, and Ar gases.

Considering the calculated traveled distances for P and Si atoms in various gases, the evaporation of P and Si in a direction normal to the melt surface could be illustrated, as shown in Figure 10. It is obvious that P atoms in the reduced pressures of H_2_ travel farther than the Si atoms. However, under He and Ar, Si atoms travel slightly farther than P. In addition, it is seen that both the P and Si atoms have shorter travel paths in Ar compared to He, which translates to higher evaporation of P and Si in the reduced pressures of He. These calculations are in good agreement with the results from the above vacuum refining trails.

As shown in Figure 2, the rate of P evaporation was the highest in the reduced pressures of H_2_ while having the lowest Si loss (Figure 3). Additionally, the reduced pressures of He in the chamber showed a higher rate of P evaporation compared to Ar, and all these cases can be explained by the illustrations in Figure 10. Finally, we propose that vacuum refining of Si under the reduced pressures of H_2_ is more efficient and accompanied with the lowest Si loss.

## Conclusions

The rate of phosphorus evaporation from liquid Si under reduced pressures of Ar, He, and H_2_ was experimentally studied and supported by studying the interactions of gas components using the kinetic theory of gases. The following remarks are highlighted as the outcomes of this research:

1. The gas-phase mass transport is a rate-controlling step for P removal from Si melt under reduced pressures, and it is affected by the type of adjacent gases of Ar, He, and H_2_.
2. For a given pressure and temperature, the kinetics of P removal is higher in H_2_ than in He and it is the lowest for Ar.
3. The resistance of the gas phase for P evaporation can be minimized via the suction of the gas close to the evaporating melt surface.
4. Apparent activation energies for the vacuum removal of phosphorus from Si in the range of 107–240 kJ/mol were determined; they are affected by the pressure and the type of gas in the vacuum chamber, and the lowest was obtained for using a low pressure of H_2_.
5. The quantum chemistry studies showed that the evaporated Si atoms have a stronger interaction with H_2_ molecules, leading to inelastic collision dynamics. The Si atoms will lose their momentum in shorter distances once they are evaporated from the melt surface, yielding a slower net evaporation of Si atoms.
6. The quantum chemistry studies indicate that the interaction of P atoms with H_2_ is mostly elastic. From the gas kinetic theory, the evaporated P atoms can travel farther away from the liquid surface in H_2_ gas than in He and Ar because of a larger number of collisions required before they lose their initial momentum. This reduces the chance of the P atoms condensing back to the liquid melt.

## Supporting Information Available

Schematic representations of the furnace and the experimental setups. Photographs of the crucibles’ cross sections after vacuum refining experiments. Photograph of the auxiliary vacuum tubes. SEM images and EDS line scans of the cross section of the molybdenum tube. The Gibbs energy for the formation of molybdenum silicides from Si_(g)_ over a wide range of temperatures (PDF).
