# Supplementary material for: Selective Vacuum Evaporation by the Control of the Chemistry of Gas Phase in Vacuum Refining of Si
Source: Langmuir. 2021 Jun 8;37(24):7473–85. doi: 10.1021/acs.langmuir.1c00876 (PMC8280733; doi:10.1021/acs.langmuir.1c00876)
Supplement: Supplementary file 1 — la1c00876_si_001.pdf [file la1c00876_si_001.pdf]

Supporting information for

Selective vacuum evaporation by control of the chemistry of gas phase in vacuum refining of Si

Arman Hoseinpur [a*], Stefan Andersson [b], Kai Tang [b], Jafar Safarian [a]

a. Department of Materials Technology, Norwegian University of Science and Technology (NTNU), 7034 Trondheim, Norway.
b. SINTEF Industry, 7465 Trondheim, Norway.
*Corresponding author's E-mail: arman.h.kermani@ntnu.no.

Table of content

- **Schematic of the furnace**
- **The pressure change in chamber**
- **Results of ICP – MS measurements**
- **Cross sections of the crucible after vacuum refining**
- **Auxiliary vacuum tubes (SEM - EDS)**

- ## Schematic of the furnace

Schematic of the vacuum furnace applied in this research is presented in Figure S1.

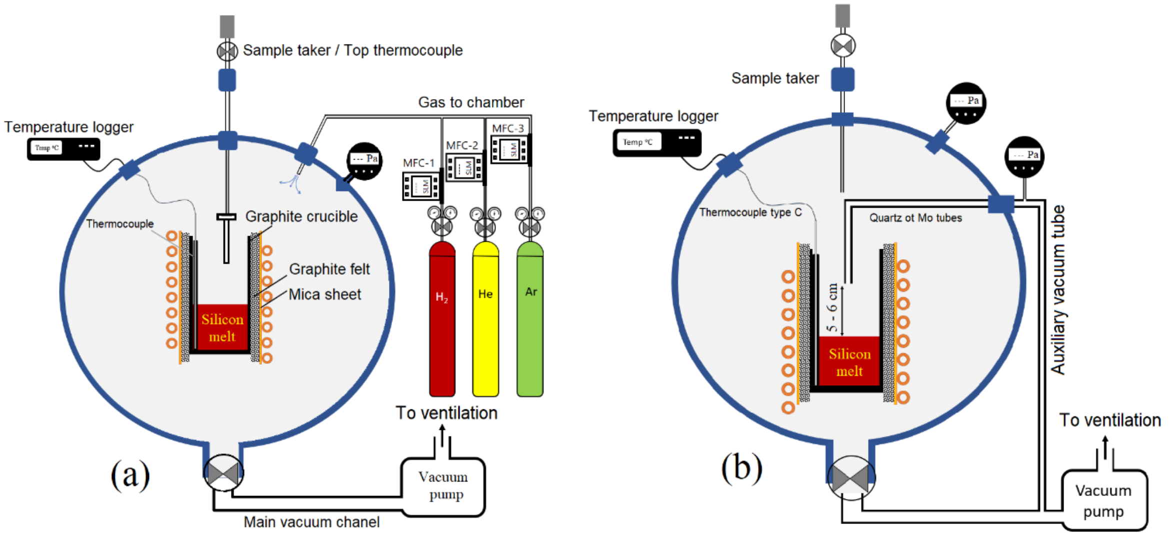

Figure S1. Schematic of the furnace and the setup configurations applied for the vacuum refining experiments. (a): the vacuum experimental setup for investigations of the effect of various gases. (b): the experimental setup for investigating the effect of applying auxiliary vacuum tubes.

- **The pressure change in chamber**

The pressure change in the vacuum refining experiment at 1650 ℃ with and without auxiliary lance is presented in the Figure S2. in the vacuum refining experiment with/without auxiliary vacuum lance

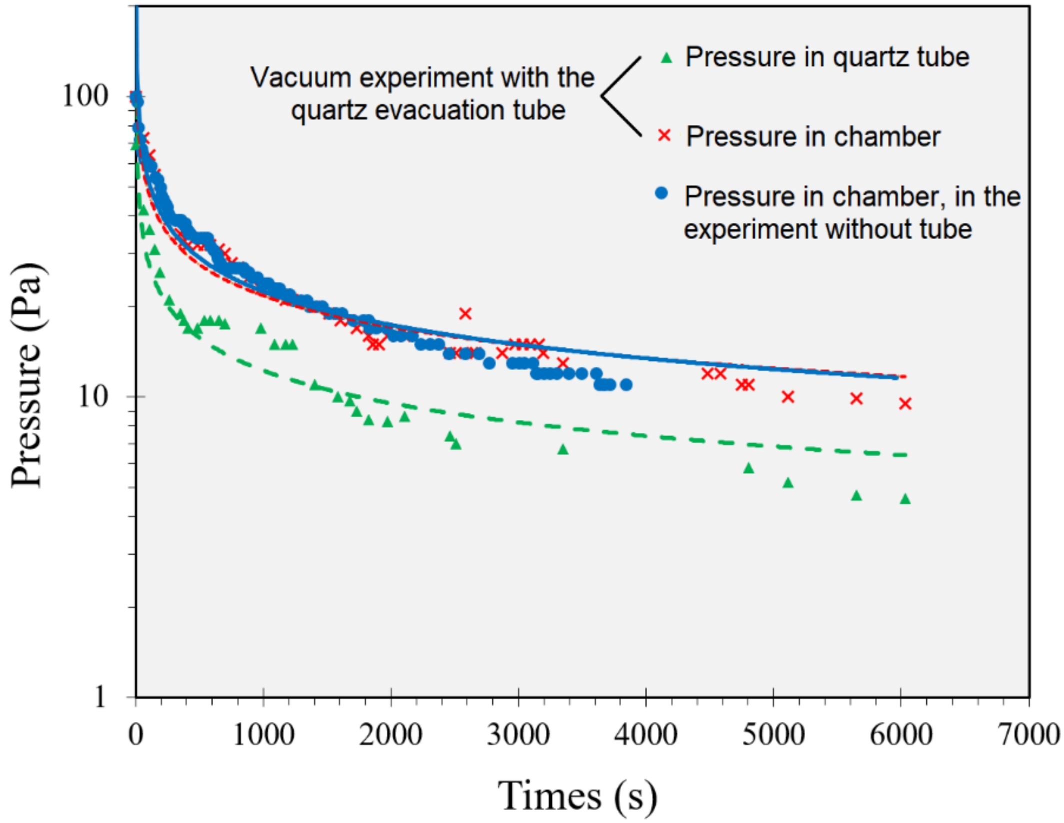

Figure S2. The measured pressures of the chamber and the vacuum tube in a vacuum experiment without the tube and the experiment with the quartz vacuum tube, both the experiments are carried out at 1650 °C.

- **Results of ICP – MS measurements**

Table S1. The phosphorus concertation in liquid Si over time of the vacuum refining experiments and the corresponding calculated k_P values from the first order kinetic model presented in the paper.

Experiment conditions	Time (minute) and phosphorus concertation (ppmw) of the melt												k_P (μm.s^{-1})	R^2
	0	10	30	35	40	60	65	80	90	120	150	160		
Vacuum, 1500 °C [3]	9.50	8.81	8.84			7.57							2.65	0.98
Vacuum, 1650 °C [3]	8.96				5.55			3.50				1.69	9.47	0.98
Vacuum, 1750 °C [3]	8.73				2.24	1.67		1.17	0.98				19.33	0.95
Vacuum with vacuum quartz tube, 1500 °C	75.80		57.55			47.05					5.58		6.03	0.98
Vacuum with vacuum quartz tube, 1650 °C	93.43				33.45			11.62					19.10	1
Vacuum with vacuum Mo tube, 1650 °C	15.07			6.34			1.70						23.20	0.97
Ar, 65 Pa, 1650 °C	9.57				9.06			8.82					0.80	0.95
Ar, 25 Pa, 1650 °C	9.64		6.87			5.69							6.89	0.97
Ar, 65 Pa, 1750 °C	11.16					9.47			8.54				2.13	0.99
Ar, 40 Pa, 1750 °C	9.85				6.25								8.36	1
Ar 25 Pa, 1750 °C	8.74				3.1			1.71		1.05			14.00	0.92
He, 65 Pa, 1650 °C	12.16		8.24			7.35							6.90	0.89
H_2, 65 Pa, 1500 °C	126.71				101.22			74.97					6.44	0.99
H_2, 25 Pa, 1650 °C	11.52		8.29			4.8			3.13				10.49	0.98
H_2, 65 Pa, 1650 °C	15.72		10.51			7.45							9.00	0.99
H_2, 65 Pa, 1750 °C	48.195		22.75			13.81							16.53	0.94

- ## Cross sections of the crucible after vacuum refining

Figure S3 shows the vertical cross-section of the graphite crucibles in 6 experiments done with various experimental conditions. As it is obvious from Figure S3 many silicon droplets are formed on the higher levels of the crucible wall. It was discussed previously [42] that there is a temperature profile in the crucible due to heat loss by radiation, and it causes Si recondensation on the top part of crucible. All the crucibles shown in Figure S3 are from experiments treated at 1650 °C, and hence they had the same temperature profile, and this indicates more Si evaporation and so recondensation. Therefore, the weight of these silicon droplets must be excluded from the final crucible weight, and hence Equation (6) plotted on Figure 6 underestimates the silicon evaporation. A comparison of all the crucibles shown in Figure 7 shows the rate of evaporation in the experiments could be arranged in descending order as: vacuum experiment with vacuum tube > vacuum experiment without the tube > experiments in 65 Pa reduced pressures > experiments with 25 Pa reduced pressures. In addition, when comparing the crucibles (c) with (d) and (e) with (f) on Figure S3, it is obvious that it had less Si evaporation and recondensation in the case of H_2 compared to He, which is in good agreement with the weight measurement results presented in Figure S3, indicating H_2 is better than He to slow down the Si evaporation.

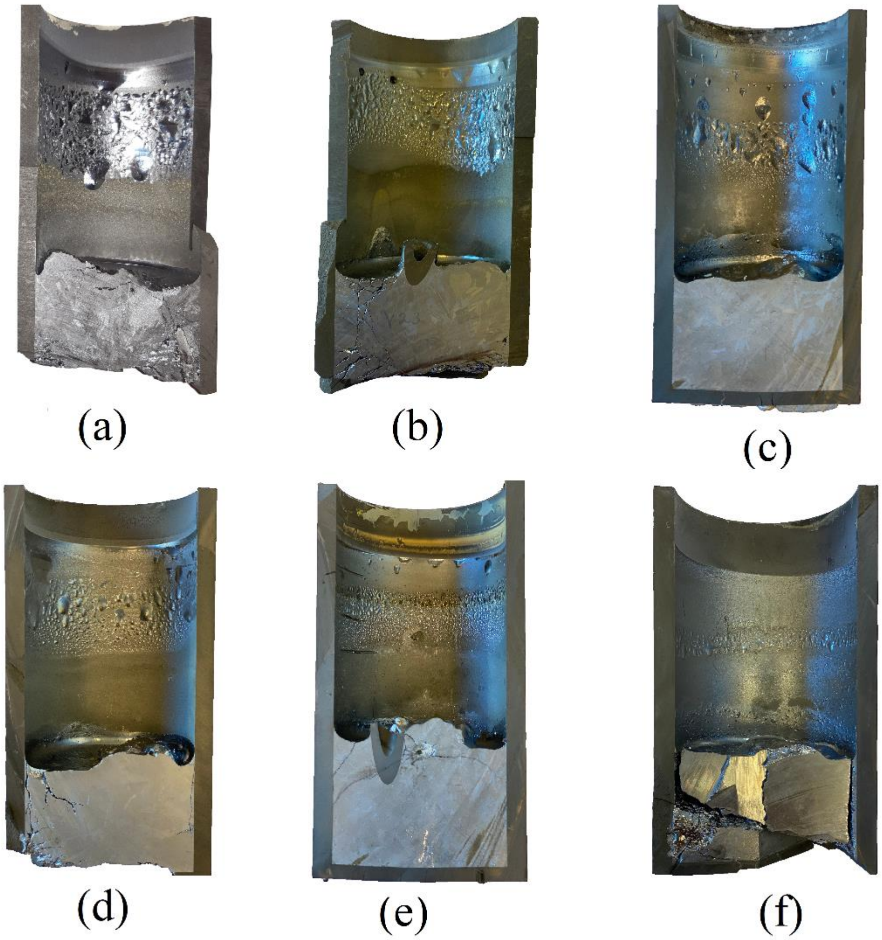

Figure S3. The photographs of the crucibles' cross sections after vacuum refining trials at 1650 ℃. (a): vacuum with quartz auxiliary vacuum tube for 80 minutes. (b): vacuum condition without the tube for 160 minutes, (c): in 25 Pa partial pressure of He after 90 minutes, (d) : in 25 Pa partial pressure of H_2 after 90 minutes, (e): in 65 Pa partial pressure of He after 90 minutes, (f): in 65 Pa partial pressure of H_2 after 90 minutes.

- **Auxiliary vacuum tubes (SEM - EDS)**

Figure S4 shows the photograph of the auxiliary vacuum tubes applied in the vacuum refining experiments. As it is obvious from Fig S4, the Mo tube is highly interacted with the Si vapors. SEM characterizations of the tip of the molybdenum tube after 65 minutes in vacuum conditions and at T = 1650 °C are presented in Figure S5 and Figure S6. Figure S5 shows the SEM micrograph of the Mo tube wall. From Figure S5, it is obvious that both the outside and inside surfaces have interacted with the Si vapors leading to the formation of a surface layer on Mo tube. Higher magnifications of the surface layer is presented in Figure S6(a) and the EDS line scan from the Mo side toward the surface is shown in Figure S6 (b & c). As can be seen from the molybdenum side toward the surface the silicon intensity increases, and there is a transition layer in between. The transition layer composition is close to the compound Mo_5Si_3 and the surface layer has a composition close to $MoSi_2$. The formation Gibbs energy of the molybdenum silicides (found in HSC 10 software data base) are calculated and presented in Figure S7. As shown on Figure S7 the mentioned molybdenum silicides are thermodynamically favorable to form.

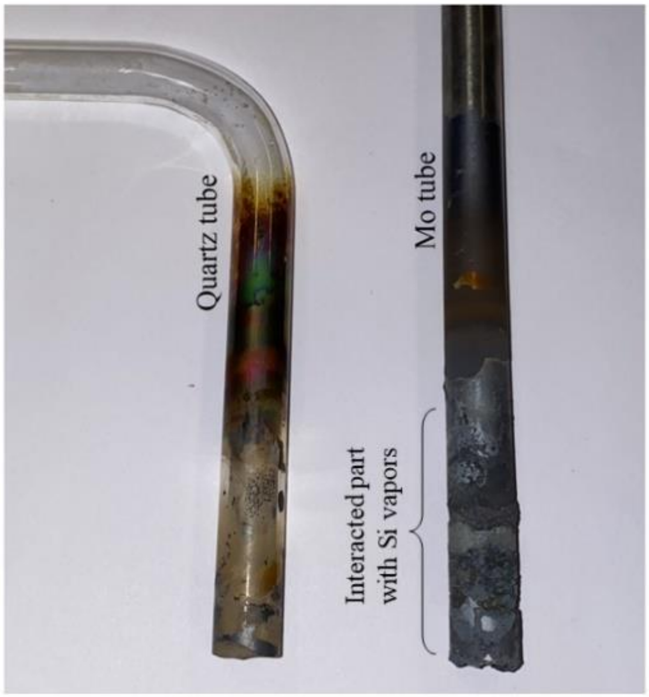

Figure S4. The photograph of the auxiliary vacuum tubes.

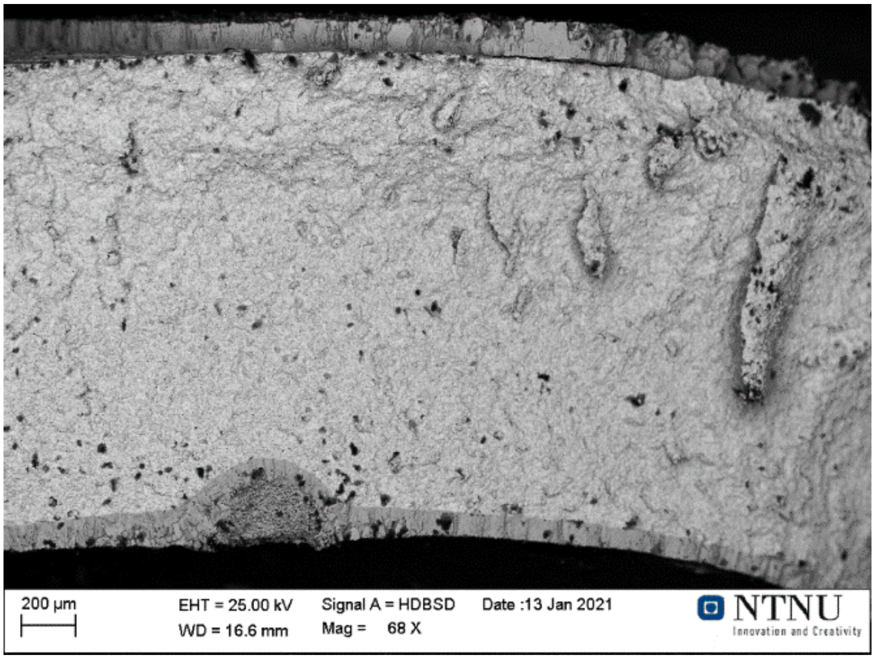

Figure S5. The SEM micrograph of the Mo tube cross-section.

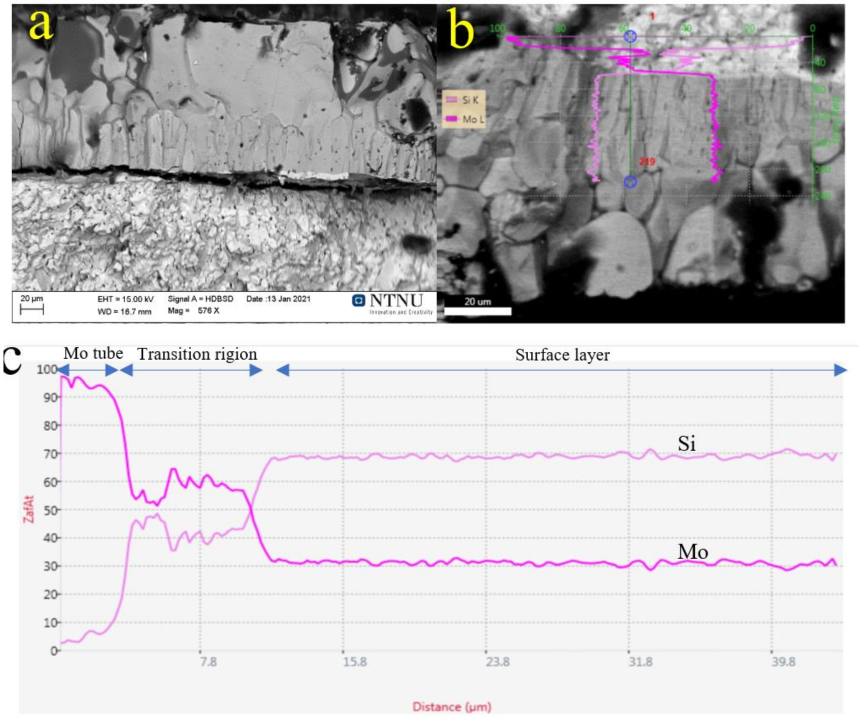

Figure S6. SEM – BSD micrographs of the Mo tubes cross-section (a & b), and the corresponding line scan (c) from Mo bilk toward to surface.

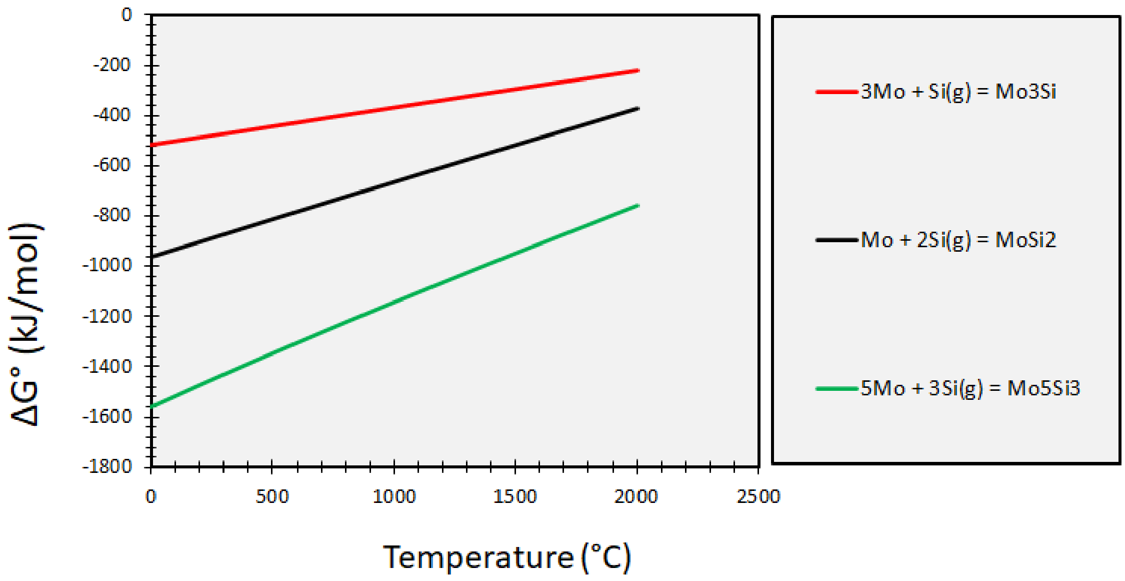

Figure S7. The Gibbs energy for the formation of the molybdenum silicides from $Si_{(g)}$ over wide range of temperatures.
